# Broadening the scope of *PLOS Biology*: Short Reports and Methods and Resources

**DOI:** 10.1371/journal.pbio.3000248

**Published:** 2019-04-26

**Authors:** The PLOS Biology Staff Editors

**Affiliations:** Public Library of Science, San Francisco, California, United States of America, and Cambridge, United Kingdom

## Abstract

This Editorial discusses the creation of two new scope-broadening article types for PLOS Biology — Methods & Resources articles and Short Reports — acknowledging that important advances in biology come in many shapes and sizes.

*PLOS Biology* is committed to transforming scientific communication to make it faster, more significant, and better connected. Our standard Research Articles have no length limits (minimum or maximum) and are intended as a catch-all for submissions; however, we’ve come to see that there is a preconceived notion that a Research Article should be a fully comprehensive “research story,” and this has been a stumbling block for all, with editors and reviewers alike wondering whether some submitted manuscripts fitted into this category. The notion of trying to wedge a square peg into a round hole was impeding our ability to consider some excellent research. We therefore wanted to better accommodate natural publishable units and output stages of the research process; having just a single standard Research Article format seemed to be limiting the studies that would make it through peer review. Acknowledging that important advances in biology come in many shapes and sizes, we revisited our criteria for publication, and back in July 2017, we launched two new article types: Short Reports and Methods and Resources.

We hope these article types will facilitate consideration of different kinds of natural research outputs and will accelerate the pace at which knowledge and information are shared and that our authors and readers will find value in them. As of April 9^th^, we’ve published 46 Methods and Resources articles and 59 Short Reports, and we encourage you to consider how your own work might fit these new formats.

The creation of new categories for these different article types allows us to expand the variety of excellent research that we can consider for publication. Yet, as our more traditional Research Articles do, our Short Reports and Methods and Resources articles adhere to our principles of high standards of quality and integrity, rigorous and fair peer-review, and expert editorial oversight.

## Short reports

We launched Short Reports because sometimes great content comes in small packages; although some scientific findings benefit from waiting until certain mechanistic details have been elucidated and confirmed by multiple experimental approaches, others are fundamentally novel and are more useful when disseminated in their early stages—even if not fully understood—so that the scientific community is free to build upon those intriguing and provocative observations. Alternatively, some studies might fit the Short Report format because they present a concise set of clever experiments that reconcile previously conflicting observations, resolve a specific conundrum, or simply apply known elegant techniques to elucidate a brief answer to an interesting scientific question.

*PLOS Biology* Short Reports are defined by novelty and interest of the phenomena being studied as well as by their format. Short Reports generally present only a limited set of experiments and should be typically summarized in 3 main figures or fewer.

The editors have revisited the Short Reports we have published since the inaugural article by Kang and colleagues [1] and would like to point our readers to some that we think are exemplary of those we seek to publish. In one example, Simonin and Roddy [2] report that during their evolution in the early Cretaceous, flowering plants were able to reduce the size of their cells by downsizing their genomes; they propose that this allowed the plants to pack more veins and stomata in their leaves and, potentially, to sustain rapid growth rates and outcompete ferns and other plants that had previously dominated terrestrial ecosystems.

In a very recent Short Report [3], Mittermeier and colleagues used more than two billion Wikipedia page searches over a period of nearly 3 years to track people’s interest in more than 30,000 species of animals and plants. As well as bringing big data to “culturomics,” the authors found that over a fifth of species showed seasonal patterns, many reflecting real-life phenology; indeed, seasonality was often more pronounced in language editions originating from countries of higher latitude. The findings are provocative and may be useful for informing conservation policy.

Finally, Lee and colleagues [4] measured dopamine release in the brains of rats while the animals performed a reward-based task in which they press a lever to get a food reward. The authors showed that simply by changing the intertrial interval, they could change the proportion of rats engaging with the lever and those engaging with the food, as well as the moment at which dopamine was released. These results reconcile conflicting findings in the literature regarding the role of phasic dopamine release in encoding prediction error and inform the understanding of between-individual differences in reward-based learning.

## Methods and Resources

We introduced Methods and Resources articles to the journal because we understand that science is in an era where inspirational technological developments abound, and large, important data sets are frequently being generated. Without an article format to accommodate these technology and/or data-focused studies, scientific discoveries could be delayed, if not prevented. There is therefore a pressing need for fully open access dissemination of innovative technologies and unique informational data sets for the intrinsic potential they offer to make new discoveries, even when the primary investigators have not yet reached that point.

Our Methods and Resources articles offer scientists the opportunity to disseminate and receive recognition for their technological innovations and/or for the generation and curation of data sets and other resources. Methods and Resources are a broad article type with 2 main subtypes. The first of these presents methodological or technological advances; such methods should be novel and show the potential to experimentally address a previously inaccessible biological question. Alternatively, the methods presented may demonstrate substantial improvements to currently used methodologies. They would need to significantly outperform their predecessors by precision, resolution, speed, accessibility, and/or cost.

The second subtype presents data sets or other scientific resources; these should be of general interest and demonstrated applicability and utility. Presented resources and data sets will be made openly available thanks to PLOS’ data policy [5], and the articles themselves are, of course, published under a CC BY license, and therefore all content is openly available for anyone to read, reuse, modify, and distribute without restriction, as long as proper attribution of authorship is maintained.

One perfect example of a Methods paper was published in our November 2017 issue; Zou and colleagues described a protocol for extracting nucleic acids from plants, animals, or microbes in less than 30 seconds and for less than $0.20 per sample [6]. Every laboratory in the world doing molecular and cellular work needs to purify nucleic acids, and the speed and simplicity of this method makes it ideally suited for both laboratory and outdoor settings, including those with limited resources such as field sites, developing countries, and teaching institutions.

And in our September 2017 issue, Cole and colleagues reported a deep whole-genome, mutagenesis-based study that identified a comprehensive set of genes relevant for plant root colonization by soil bacteria [7]. In addition to providing important new insights into a fundamental interspecific interaction, this study also created a valuable resource for deciphering gene function and enriching genome annotations. Another valuable resource is provided by Zhang and colleagues, who sequenced and annotated the genome of the Japanese sea cucumber. Analysis of the genome revealed insights into the origin of chordates and into the regenerative potential of echinoderms [8].

## Evolving article types

We’re evolving *PLOS Biology* and have other new article types planned for this year. Send us your suggestions for other formats, and we’ll gladly consider them. And—as always—we welcome your submissions based on these guidelines. If you’ve already published a Short Report or a Methods and Resources article with *PLOS Biology*, we encourage you to build upon your initial observation, to use your recently developed technique, or to further investigate the data sets you’ve produced to generate novel mechanistic insights in biology and to submit your further advances to our journal.
